# Hatha yoga is more effective in improving kinesiophobia and subjective well-being when combined with self-compassion meditation in people with CLBP: a randomized control trial

**DOI:** 10.3389/fpsyg.2023.1272919

**Published:** 2023-11-20

**Authors:** Andrea De Giorgio, Sonia Angilletta, Barbara Matteo, Valerio Bonavolontà, Nicola Luigi Bragazzi, Goran Kuvačić

**Affiliations:** ^1^Faculty of Psychology, eCampus University, Novedrate, Italy; ^2^Klinikos Center for Psychodiagnostics and Psychotherapy, Rome, Italy; ^3^Department of Applied Clinical Sciences and Biotechnology, University of L’Aquila, L’Aquila, Italy; ^4^Laboratory for Industrial and Applied Mathematics, Department of Mathematics and Statistics, Faculty of Science, York University, Toronto, ON, Canada; ^5^Faculty of Kinesiology, University of Split, Split, Croatia

**Keywords:** emotions, fear of movement, spine, psychological factors, chronic low back pain

## Abstract

**Objective:**

The present study aims to examine whether combining hatha yoga practice with self-compassion meditation could influence kinesiophobia, emotions, perceived stress, and perceived disability among individuals with chronic low back pain when compared with hatha yoga alone.

**Methods:**

The randomized controlled study included 70 participants with chronic low back pain (CLBP) who were randomly assigned to a Hatha yoga group (HY; n = 35) or a Hatha yoga group plus self-compassion meditation (HYSCM; n = 35). Participants followed a protocol for 8 weeks, and the assessments were carried out before and after the intervention, with a follow-up evaluation conducted after one month. The participants completed the PANAS, PSS, TSK, and RMQ questionnaires. A Generalized Estimating Equation was used to explore the effect of interventions.

**Results:**

Both HY and HYSCM groups improved kinesiophobia, perceived disability, and stress at the follow-up. However, the HYSCM group shown a more significant reduction in kinesiophobia compared to the HY group. Moreover, significant improvement in positive affect and a reduction in negative affect over time in the HYSCM group were observed.

**Conclusion:**

Hatha yoga practice when combined with self-compassion meditation led to extra benefits by exhibiting a prolonged effect, especially on kinesiophobia positive and negative effects.

## 1 Introduction

Low back pain is described as a pain syndrome in the lower back pain region, typically categorized by its duration: (i) acute, when pain lasts less than six weeks; (ii) sub-chronic, when it persists for six-twelve weeks; (iii) chronic, when pain persists more than twelve weeks, referred to as chronic low back pain (CLBP; Indahl, 2004; Van Zundert et al., 2013). CLBP is a widespread health problem and is the most common among painful musculoskeletal conditions, making it one of the costliest medical issues (Cypress, 1983; Russo et al., 2018). Indeed, both low back pain and its chronic form are currently the leading cause of disability globally (Vos et al., 2015). The epidemiology and prevalence of these conditions are well documented in the literature (Kent and Keating, 2005; Freburger et al., 2009). It is estimated that 85% of back pain diagnoses are related to non-specific vertebral-mechanical disorders (Ledford, 2017; Will et al., 2018). There are many potential causes of CLBP and the differential diagnosis is challenging, the origin of this condition is primarily attributed to two main factors: i) mechanical-degenerative origin (Furunes et al., 2017; Will et al., 2018) which can be identified through imaging (Sabnis et al., 2017) and functional impairment (Delitto et al., 2012; Will et al., 2018); ii) non-mechanical origin which may result from alterations in brain structure and function (Wand et al., 2011).

The literature emphasizes that the symptomatology of CLBP can be intensified by psychological factors (Petrucci et al., 2021). Among these, the general quality of life and, in particular, emotions, can affect posture and body signal awareness (Dael et al., 2012; De Giorgio et al., 2017a,b, 2018a,b; Viglino et al., 2022), thereby compromising the perception of the CLBP (Crofford, 2015). Among them, anxiety play a pivotal role in the experience of pain because they exacerbate the painful sensation leading to avoidance behaviors, a phenomenon known as kinesiophobia (Padovan et al., 2018, 2019). Negative affect and kinesiophobia interact each other, creating a lasting vicious circle that results in physical and psychosocial disability (Trocoli and Botelho, 2016; Madsen et al., 2022). Studies have shown that individuals with CLBP who experience more negative affect than positive ones have lower levels of health-related quality of life (Staes et al., 2007), and vice-versa (Lysne et al., 2021). Furthermore, literature indicates a correlation between kinesiophobia, pain, proprioception, and functional performance (Asiri et al., 2021), demonstrating its pivotal role in the etiology of CLBP and associated disability (Fritz et al., 2001).

Among the interventions that have been shown to be effective both psychologically and physically are those that include yoga. Yoga training interventions have been proven to improve spinal flexibility in people affected by CLBP (Tekur et al., 2008), both in elderly women and men (Brems, 2015; Buttner et al., 2015). Such interventions improve the physical status by stimulating the release of various hormones related to bodily well-being (Sherman et al., 2013; Lee et al., 2014) and brain-derived neurotrophic factor, suggesting that the potential decrease in pain level may arise from yoga exercise (Posadzki and Ernst, 2011). Furthermore, yoga has proved beneficial in improving psychological indices (Davis et al., 2015) and stress (Brems, 2015). However, emerging evidence highlights that the mechanisms behind these results are still unclear, necessitating further research.

In general, mindfulness-based interventions have proven both a role in stress (Pascoe et al., 2017); also in work, (Ramaci et al., 2020) and effective in reducing chronic pain (Reiner et al., 2013) while simultaneously enhancing quality of life and psychological indices among chronic pain sufferers, including those with CLPB (Cramer et al., 2013; La Cour and Petersen, 2015). Recently, it has been demonstrated that self-compassion meditation (SCM) can alter the neural response to evoked pain in CLBP people (Berry et al., 2020). SCM differs substantially from other mindfulness approaches. While the latter focus on inner experiences such as sensations, emotions, and thoughts, SCM is aimed at the individual experiencing suffering (Neff and Germer, 2013). In the context of CLBP, mindful awareness is directed toward acknowledging body sensations like as pain in all its forms (e.g., stabbing, burning quality) in a non-judgmental way, while SCM is specifically designed and developed to emphasize soothing and comforting the “self” when discomforting and distressing experiences, reminding us that such experiences are part of being human (Neff and Germer, 2013).

Therefore, the main aim of this study was to determine if Hatha yoga is more effective when combined with self-compassion meditation in reducing kinesiophobia and improving the subjective well-being in individuals with CLBP. Specifically, we attempted to combine a Hatha yoga protocol with a self-compassion meditation in order to investigate whether SCM can influence the kinesiophobia, positive/negative affect, perceived stress, and level of perceived low back disability.

## 2 Materials and methods

### 2.1 Participants

Seventy participants were enrolled by two general practitioners based on their medical history and the cohort consisted of 47 males and 23 females with a mean age of 35.86 ± 7.36 years. They were randomly and blindly assigned to Hatha yoga group (Control or HY; 23 males and 12 females with a mean age of 35.14 ± 7.36 years) or to Hatha yoga plus Self-compassion meditation (Experimental or HYSCM; 24 males and 11 females with a mean age of 36.57 ± 6.94 years) using simple randomizing approach. To create the random number sequence, an independent researcher, utilized the Random Number Generators feature in SPSS 28.0 statistical software. The resulting sequence was then securely stored in sealed, numbered envelopes. As participants were enrolled, a research assistant systematically opened these envelopes in order and assigned participants to their respective groups. Allocation was determined based on the order of enrollment.

**FIGURE 1:**
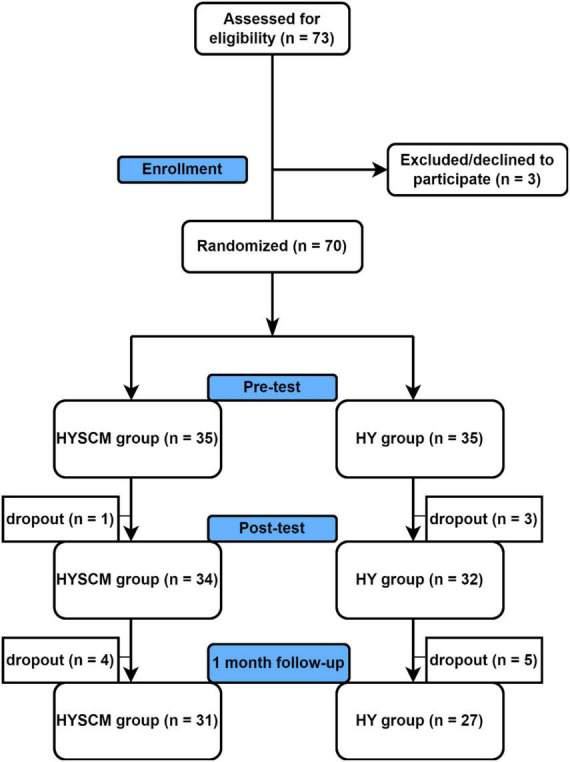
Recruitment and participant flow chart. Figures were made ad hoc.

Inclusion criteria were: (i) persistent CLBP; (ii) adult age (≥18 years old). Exclusion criteria were: (i) acute low back pain including recent thoracic-lumbar trauma; (ii) specific causes of low back pain such as lumbar stenosis, disk hernia, spinal deformity, fracture, spondylosis, osteoporosis of the spine; iii) current or pre-existing neurologic, oncologic, or psychiatric conditions (e.g., dementia, Parkinson’s disease, congenital central nervous system malformations, multiple sclerosis, tumors, schizophrenia, head trauma) as reported by general practitioners; iv) individuals with recent cerebrovascular accidents and myocardial infarctions, as reported by general practitioners. A power analysis using free software (G*Power) (Faul et al., 2007) was conducted to determine an adequate sample size for the study. The analysis revealed that a minimum of 62 participants is needed, calculated based on a study power of 80%, α set at 0.05, and an effect size of 0.3. With anticipation of a possible dropout rate of 10%, it was decided that each group should have 35 participants. Following consent to participate, HY and HYSCM classes were provided to all the participants (Figure 1). During the post-test phase, one participant (3%) from the experimental group withdrew, resulting in a sample of 34 participants included in the post-test analysis. Three participants (8%) withdrew from the control group, leaving a sample of 32 participants for post-test analysis. In the follow-up phase, four participants (11%) from the experimental group and seven participants (20%) from the control group withdrew, resulting in final samples of 31 and 27 participants, respectively.

All participants voluntarily participated in the study. They each provided written consent after receiving comprehensive information about the study’s objectives, advantages, and possible risks. Consent forms were specifically approved by the “The Ethical Committee of the Faculty of Kinesiology” (Split, Croatia). This committee approved the entire study design, which was conducted according to the ethical standards of the 1964 Helsinki Declaration and its subsequent amendments (2181-205-02-05-23-025).

### 2.2 Procedures

**FIGURE 2:**
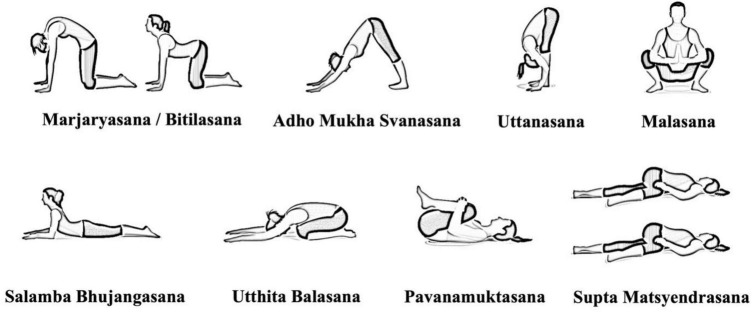
Asanas chosen by the certified yoga instructor.

Participants in the HY group attended only the yoga sessions (1 h, 3 days per week, over 8 weeks) while the HYSCM participated in both the yoga sessions and an additional self-compassion meditation (totaling 1 h 30 min, 3 days per week, over 8 weeks). All sessions took place under the same conditions (room, light, and temperature ≈23°C) and were led by the same professional with a master’s degree holder in clinical psychology, certified yoga, MBSR-MBCT instructor, and expert in self-compassion meditation. Before starting the sessions, the instructor conducted a preliminary “zero session” to familiarize all participants with the practices to be conducted, explaining and giving them a chance to try them out. This lesson was critical in making participants aware of the kind of effort required and how their spine was responding to asana practices. The Hatha yoga sessions, identical for both groups, lasted an hour and included the following activities:

• *Sama vritti pranayama* (10 min.): This is a breathing practice characterized by a one-to-one ratio respiratory rate. Specifically, we employed a pattern of inhaling and exhaling to the count of four: inhale for 4 s; hold the breath in for 4 s; exhale for 4s; hold the breath out for 4s; then restart the cycle;
• *Asanas* (40 min; Figure 2): Three series of eight asanas were performed, specifically: Marjaryasana/Bitilasana (Cat/Cow Pose); Adho Mukha Svanasana (Downward-Facing Dog Pose); Uttanasana (Standing Forward Bend); Malasana (Garland Pose); Salamba Bhujangasana (Sphinx Pose); Utthita Balasana (Extended Child’s); Pavanamuktasana (Wind-Removing); Supta Matsyendrasana (Reclining Spinal Twist).

The instructor emphasized the importance of not overexerting oneself while performing the asanas and encouraged participants to perform them to the best of their abilities. So, while the instructor maintained for three breaths to five deep breaths (or about 1 min) according to the specific asana, participants could occupy the time of each asana according to their ability and physical possibilities. Pause were set between each asana and between each series.

• *Yoga Nidra* (10 min.): The final relaxation technique was characterized by the rotation of body awareness, breath awareness, and awareness of feelings and sensations. In our study the Sankalpa, i.e., intentions, and final visualization were not included.

The 30-min self-compassion meditation was performed only by HYSCM group and was characterized by three components: self-kindness versus self-judgment; common humanity versus isolation; and mindfulness versus over-identification. Participants meditated on each component for 10 min.

### 2.3 Outcome measures

Psychological assessments were provided to the participants before (pre-) and after (post-) the intervention. Several questionnaires were included related to: i) psychological affective experiences (PANAS, *Positive and Negative Affect Schedule;* Terracciano et al., 2003); ii) perceived stress (PSS, *Perceived Stress Scale*; Mondo et al., 2021); iii) fear of movement (i.e., kinesiophobia; TSK, *Tampa Scale of Kinesiophobia*; Burwinkle et al., 2005); iv) level of perceived disability (RMQ, R*oland Morris Low Back Pain and Disability Questionnaire;* Padua et al., 2002). Participants from both the HY and HYSCM groups received and completed the questionnaires before the first Hatha yoga session [and self-compassion meditation] (pre-evaluation) and immediately after the last Hatha yoga session [and self-compassion meditation] (post-evaluation). A follow-up evaluation was provided after one month after post-evaluation. The scoring of the questionnaires is reported as follows:

• *PANAS*: This questionnaire evaluates positive and negative affect using 20-items that reflect the most general dimensions of individual affective experiences. It comprises a series of words describing different feelings and emotions, each assessed on a Likert scale indicating the extent to which the participant has felt a particular state over the past week, with scores ranging from 1 (not at all/very little) to 5 (extremely). Positive Affect Score (PA) is calculated by adding the scores on items 1, 3, 5, 9, 10, 12, 14, 16, 17, and 19. Scores can range from 10 – 50, where higher scores representing higher levels of positive affect. The Negative Affect (NA) Score is derived from the sum of the scores on items 2, 4, 6, 7, 8, 11, 13, 15, 18, and 20, with scores ranging from 10 to 50, and lower scores indicating lower levels of negative affect.
• *PSS*: This 10-item questionnaire evaluates perceived stress and is among the most widely used tools to measure this psychological experience. Responses are given on a Likert scale from 0 (never) to 4 (very often). The score is obtained by reversing the responses (e.g., 0 = 4, 1 = 3, 2 = 2, 3 = 1, and 4 = 0) given to the four positively worded items (items 4, 5, 7, and 8) and then summing all scale items.
• TSK: Kinesiophobia: This instrument evaluates the fear of movement and consists of 13 items scored on a four-point Likert scale from 0 (strongly disagree) to 4 (strongly agree). In the Italian version (Monticone et al., 2010) no cut-off was established, and it was recommended to consider the total score.
• RMQ: This instrument evaluates the perceived disability caused by low back pain. It is scored by adding up the number of items checked by the patient in a 24-item questionnaire (Padua et al., 2002). Total score ranges from 0 to 24, where 0–9 indicates low disability, 10–13 indicates mid disability, up to 14 points indicate severe disability.

### 2.4 Statistical analysis

Descriptive statistics, including means and standard deviations (SD), frequencies, or percentages (%), were used to summarize the socio-demographic and clinical variables, based on their nature. Group comparisons of these variables were conducted using Student’s independent t-test for continuous variables and the Chi-Square (χ^2^) test for categorical data (with Fisher’s test used when necessary). The normality of the distribution of continuous variables was confirmed by the Shapiro-Wilk test. A Generalized Estimating Equation (GEE) on an intention to treat basis (ITT) was used to explore the effects of the intervention on TSK, PSS, RMQ, PA and NA by using group and time as main effects in three different time points. Liang and Zeger (1986) introduced the GEE methodology to accurately estimate the regression coefficient and its variance when correlated data is utilized in regression analyses. The GEE model is a robust method for analyzing longitudinal data that may not be normally distributed and have variable variances of outcome measures. Moreover, this model is also designed to accommodate missing data which is a common issue in randomized controlled trials (RCTs). Whenever a group × time interaction effect was observed, a pairwise comparison with Bonferroni correction was conducted to compare the differences between the intervention groups. To estimate the effect size for the group-by-time interaction, Cohen’s d was calculated by comparing the change scores with a t-test and converting the t-statistic using the formula d = 2(t)/sqrt (df), where df represents the degrees of freedom. The magnitude of d was qualitatively interpreted using the following thresholds (Hopkins et al., 2009): < 0.2, trivial; 0.2 to 0.6, small; 0.6 to 1.2, moderate; 1.2 to 2.0, large; and 2.0 to 4.0, very large. A significance level of p < 0.05 was chosen for the analysis. The data were analyzed using SPSS 28.0 statistical software (SPSS, Chicago, IL, USA) and GraphPad Prism 9 (GraphPad Software, Inc., San Diego, CA, USA).

## 3 Results

### 3.1 Clinical and demographic characteristics of the participants

The clinical and demographic characteristics of the participants at the baseline are presented in Table 1. Most of them were males (67%) and employed (66%). Moreover, most participants (64%) had a high school diploma, while only three participants (4%) had completed elementary school. Only 20% of the entire sample were single. Regarding the participants’ previous experience with yoga and meditation, most of the sample reported having no prior experience with these practices. Specifically, 73% of participants (n = 51) reported no previous yoga experience, while 81% (n = 57) reported no previous meditation experience out of the total sample of 70 participants. The results of the chi-squared and t-tests indicated that there were no significant differences in all variables at baseline between the two groups (*p* > 0.05). These findings suggest that no potentially confounding variables were present in the initial group assignment, which strengthens the validity of the subsequent analyses.

**TABLE 1:** Demographic and clinical characteristics of participants at baseline.

Variable	HYSCM (*n* = 35)		HY (*n* = 35)		χ^2^/t	*p* values
Age, mean (SD)	36.57	(6.94)	35.14	(7.80)	0.809	0.421
Sex, n (%)					0.065	0.799
Male	24	(69)	23	(66)		
Female	11	(31)	12	(34)		
BMI, mean (SD)	24.94	(3.19)	25.49	(3.11)	−0.727	0.470
Occupation, *n* (%)					6.246	0.1
Employed	15	(43)	12	(34)		
Self-employed	6	(17)	13	(37)		
Domestic work	9	(26)	3	(9)		
Unemployed	5	(14)	7	(20)		
Education, *n* (%)					2.214	0.529
Elementary school	1	(3)	2	(6)		
Middle school	4	(11)	1	(3)		
High school	22	(63)	23	(66)		
University diploma or higher	8	(23)	9	(25)		
Marital status, *n* (%)					0.654	0.721
Single	8	(23)	6	(17)		
Engaged	12	(34)	15	(43)		
Married	15	(43)	14	(40)		
Pharmacological therapy, *n* (%)					1.628	0.443
Analgesic	14	(40)	12	(34)		
Muscle relaxant	10	(29)	15	(43)		
Both	11	(31)	8	(23)		
Previous yoga therapy, *n* (%)					0.072	0.788
Yes	9	(26)	10	(29)		
No	26	(74)	25	(71)		
Previous mediation experience, *n* (%)					0.094	0.759
Yes	7	(20)	6	(17)		
No	28	(80)	29	(83)		
Smoking, n (%)					3.66	0.056
Yes	22	(63)	14	(40)		
No	13	(37)	21	(60)		

Legend: Means with standard deviations (SD); number (n) of cases with percentages (%); χ^2^/t – chi square/t statistics value; BMI, body mass index.

### 3.2 Effects of hatha yoga interventions

**FIGURE 3:**
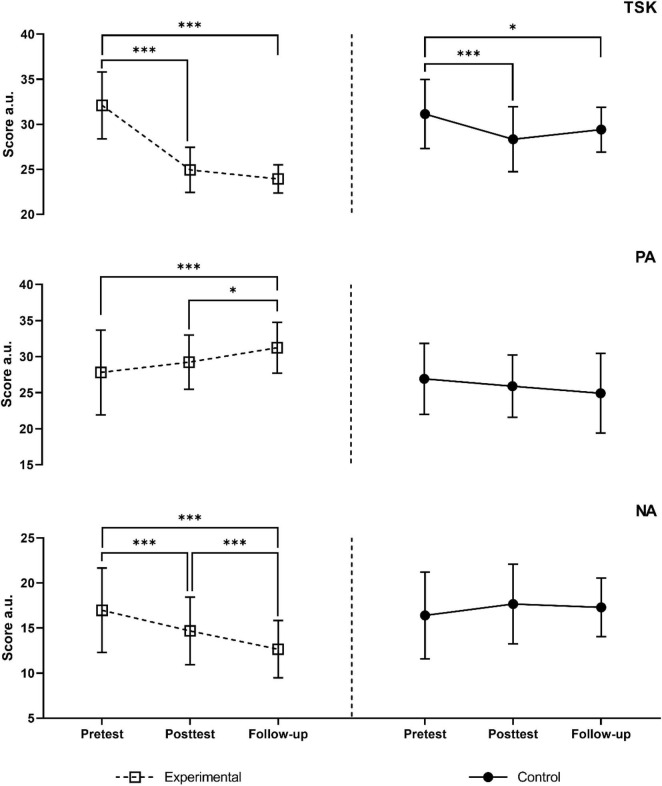
Changes in TSK, PA, and NA scores at different time points; *p < 0.05; ***p < 0.001.

The findings of the Generalized Estimating Equation (GEE) analysis are presented in Table 2. The results indicate that significant group × time interaction effects in GEE model were observed for the TSK (Wald χ^2^ = 141.51, *p* = 0.047), PA (Wald χ^2^ = 3.07, *p* = 0.047), and NA (Wald χ^2^ = 23.51, *p* = 0.047) variables. Accordingly, *post hoc* analyses were conducted on these variables. As presented in Table 2, the GEE model showed no significant differences between the two groups in TSK at pre-test. The results of the group × time interaction analysis indicated that there were statistically significant differences in the change of TSK from pre-test to post-test (Wald χ^2^ = 24.08, *p* < 0.001, ES = 1.2 [large]) and follow-up (Wald χ^2^ = 47.32, *p* < 0.001, ES = 1.499 [very large]) between the groups. This suggests that the HYSCM group had a greater decrease in TSK from pre-test to post-test and one-month follow-up, by 4.42 and 6.10 points, respectively, compared to the HY group. The within-group analysis showed that both groups had a significant decrease in TSK from pre-test to post-test (*p* < 0.001), and pre-test to follow-up (HYSCM: *p* < 0.001; HY: *p* = 0.026) as presented in Figure 3 and Table 3.

**TABLE 2:** Effects of Hatha + SCM intervention on the participants’ TSK, PSS, RMQ, PA, and NA at pre-test, post-test, and follow-up.

Variable	B	SE	95% CI		Wald χ^2^	*p*	ES
			lower	upper			
**TSK**							
Intercept	31.14	0.64	29.89	32.40	2374.84	<0.001	
Group (HYSCM)*^a^*	0.97	0.89	−0.77	2.71	1.19	0.274	
Time (post-test)*^b^*	−2.77	0.62	−3.99	−1.54	19.60	<0.001	
Time (follow-up)*^b^*	−2.04	0.65	−3.32	−0.76	9.80	0.002	
Group (HYSCM) x time (post-test)*^c^*	−4.42	0.90	−6.19	−2.66	24.08	<0.001	1.2
Group (HYSCM) x time (follow-up) *^c^*	−6.10	0.89	−7.84	−4.36	47.32	<0.001	1.499
**PSS**							
Intercept	13.57	0.84	11.92	15.22	259.77	<0.001	
Group (HYSCM)*^a^*	−0.69	1.17	−2.99	1.62	0.34	0.559	
Time (post-test)*^b^*	−1.10	0.61	−2.29	0.09	3.29	0.070	
Time (follow-up)*^b^*	0.51	1.10	−1.64	2.66	0.21	0.645	
Group (HYSCM) x time (post-test)*^c^*	−0.54	0.81	−2.13	1.05	0.44	0.508	0.13
Group (HYSCM) x time (follow-up) *^c^*	−3.54	1.51	−6.50	−0.58	5.48	0.019	0.636
**RMQ**							
Intercept	15.03	0.70	13.66	16.40	463.47	<0.001	
Group (HYSCM)*^a^*	−0.80	1.09	−2.94	1.34	0.54	0.464	
Time (post-test)*^b^*	−3.87	0.79	−5.41	−2.33	24.25	<0.001	
Time (follow-up)*^b^*	−3.14	0.92	−4.94	−1.35	11.77	<0.001	
Group (HYSCM) x time (post-test)*^c^*	−0.33	1.11	−2.52	1.85	0.09	0.765	0.044
Group (HYSCM) x time (follow-up)*^c^*	−0.19	1.31	−2.75	2.37	0.02	0.885	0.017
**PA**							
Intercept	26.91	0.82	25.31	28.52	1075.93	<0.001	
Group (HYSCM)*^a^*	0.89	1.28	−1.62	3.39	0.48	0.488	
Time (post-test)*^b^*	−0.86	0.96	−2.75	1.03	0.80	0.371	
Time (follow-up)*^b^*	−1.93	1.24	−4.37	0.51	2.41	0.121	
Group (HYSCM) x time (post-test)*^c^*	2.41	1.12	0.22	4.60	4.66	0.031	0.521
Group (HYSCM) x time (follow-up)*^c^*	5.41	1.51	2.46	8.36	12.91	<0.001	0.829
**NA**							
Intercept	16.40	0.80	14.82	17.98	415.80	<0.001	
Group (HYSCM)*^a^*	0.57	1.12	−1.63	2.77	0.26	0.610	
Time (post-test)*^b^*	1.26	0.81	−0.33	2.87	2.41	0.121	
Time (follow-up)*^b^*	1.27	0.91	−1.22	2.35	0.39	0.534	
Group (HYSCM) x time (post-test)*^c^*	−3.64	0.92	−5.44	−1.84	15.67	<0.001	0.995
Group (HYSCM) x time (follow-up)*^c^*	−5.00	1.11	−7.19	−2.82	20.11	<0.001	1.043

Summary statistic from generalized estimating equations model (GEE) for all variables (*n* = 70).

^*a*^Group effect is defined as the between-group difference between the HYSCM and HY groups at pre-test.

^*b*^Time effect is defined as the magnitude of change in the control group at both the post-test and one month follow-up compared to the pre-test.

^*c*^Group*time effect defined as the group difference between the HYSCM and HY groups in the magnitude of change in scores at post-test and one month follow-up relative to pre-test B, partial regression coefficients; SE, standard error; 95% CI, 95% confidence interval; Wald χ2, Wald Chi Square value; p, probability value; ES, effect size calculated by determining the difference in mean change between the two groups and pooled baseline SD.

**TABLE 3:** Within-group comparison of changes in TSK, PA, and NA scores at different time points.

Variable	Time point	Group	Adjusted mean difference	(SE)	95% CI		Bonferroni
					Lower	Upper	*post hoc*
**TSK**							
	Pre-test / Post-test	HYSCM	−7.19	0.65	−9.09	−5.28	Post-test < Pre-test
		HY	−2.77	0.62	−4.60	−0.93	Post-test < Pre-test
	Pre-test / Follow-up	HYSCM	−8.15	0.60	−9.91	−6.38	Follow-up < Pre-test
		HY	−2.04	0.65	−3.96	−0.13	Follow-up < Pre-test
	Post-test / Follow-up	HYSCM	−0.96	0.35	−1.99	0.07	
		HY	0.72	0.45	−0.59	2.03	
**PA**							
	Pre-test / Post-test	HYSCM	1.54	0.56	−0.11	3.20	
		HY	−0.86	0.96	−3.69	1.96	
	Pre-test / Follow-up	HYSCM	3.48	0.85	0.99	5.97	Follow-up < Pre-test
		HY	−1.93	1.24	−5.58	1.72	
	Post-test / Follow-up	HYSCM	1.94	0.65	0.04	3.83	Follow-up < Post-test
		HY	−1.07	0.90	−3.71	1.57	
**NA**							
	Pre-test / Post-test	HYSCM	−2.37	0.42	−3.60	−1.14	Post-test < Pre-test
		HY	1.27	0.82	−1.13	3.67	
	Pre-test / Follow-up	HYSCM	−4.44	0.65	−6.34	−2.54	Follow-up < Pre-test
		HY	0.56	0.91	−2.10	3.23	
	Post-test / Follow-up	HYSCM	−2.07	0.49	−3.51	−0.63	Follow-up < Post-test
		HY	−0.71	0.87	−3.25	1.84	

SE, standard error; 95% CI, 95% confidence interval.

There were no significant differences between the two groups in PSS at pre-test. Significant group × time interaction effects were observed at follow-up (Wald χ^2^ = 5.48, *p* = 0.019, ES = 0.636 [moderate]), but not at post-test (Wald χ^2^ = 0.44, *p* = 0.508, ES = 0.13 [small]). This suggests that the effect of the intervention on perceived stress differed between the HYSCM and HY groups over time, with the HYSCM group showing a greater reduction in perceived stress at follow-up compared to the HY group. Significant interaction effect at follow-up only suggests that the intervention may have had a delayed effect on reducing perceived stress.

Regarding the RMQ the effect of group was not statistically significant (Wald χ^2^ = 0.54, *p* = 0.464). However, significant effects were observed for time at post-test (Wald χ^2^ = 24.25, *p* < 0.001) and follow-up (Wald χ^2^ = 11.77, *p* < 0.001). Moreover, a non-significant group × time interaction effect was found at post-test (Wald χ^2^ = 0.09, *p* = 0.765, ES = 0.044 [trivial]) and follow-up (Wald χ^2^ = 0.02, *p* = 0.885, ES = 0.017 [trivial]). This suggests that the effect of the intervention on disability did not differ between the HYSCM and HY groups over time. Significant time effects were observed at both post-test and follow-up, indicating that disability decreased over time regardless of group assignment.

The GEE model showed no significant differences between the two groups in PA at pre-test (Wald χ^2^ = 0.48, *p* = 0.488). Significant group x time interaction effects were observed at both post-test (Wald χ^2^ = 4.66, *p* = 0.031, ES = 0.521 [small]) and follow-up (Wald χ^2^ = 12.91, *p* < 0.001, ES = 0.829 [moderate]). This suggests that the effect of the intervention on positive affect differed between the HYSCM and HY groups over time. Specifically, at post-test, the HYSCM group had a greater increase in positive affect (on average by 2.41 points), and at follow-up, (on average by 5.41 points) compared to the HY group. Moreover, the *post hoc* analysis revealed the same trend in changes for HYSCM group (Figure 3 and Table 3).

Considering NA, no significant differences between the two groups in NA at pre-test Wald χ^2^ = 0.26, *p* = 0.610) were found. A statistically significant differences were observed between the groups in terms of the change from pre-test to post-test (Wald χ^2^ = 15.67, *p* < 0.001, ES = 0.995 [moderate]) and follow-up (Wald χ^2^ = 20.11, *p* < 0.001, ES = 1.043 [moderate]), according to the analysis of group x time interaction results. Over time, there seemed to be a difference in the effect of the intervention on NA between the HYSCM and HY groups, where at post-test, the HYSCM group had a decrease in NA (on average by 3.64 points) compared to the HY group. The same was observed at follow-up, where the HYSCM group had a decrease in NA (on average by 5.00 points) compared to the HY group. Within-group analysis revealed significant changes from pre-test to post-test (p < 0.001), pre-test to follow-up (*p* < 0.001), and post-test to follow-up (*p* < 0.001) for the HYSCM group, whereas no significant changes were observed for the HY group.

## 4 Discussion

In the present study, the effects of Hatha Yoga and a combined approach of Hatha Yoga and Self-Compassion Meditation on measures of kinesiophobia, positive and negative affect, perceived stress, and level of perceived low back disability in people with chronic low back pain were investigated.

Both groups showed significant improvements in kinesiophobia from pre-test to post-test and follow-up. However, the improvements were significantly greater in the HYSCM group. This may suggest that the addition of self-compassion meditation helped participants to confront their fear of movement more effectively. Incorporating self-compassion meditation, as a potential aid in reducing kinesiophobia and influence the chronic pain, is consistent with the literature that emphasizes the significance of psychological factors in pain management and the potential advantages of mindfulness-based approaches in decreasing fear-avoidance beliefs and behaviors linked to chronic pain (Wren et al., 2012; Veehof et al., 2016). For example, the cognitive-behavioral models of chronic pain conceptualize the etiology of pain as a vicious circle in which fear of pain initiates avoidance behavior, which contributes to deconditioning, and may maintain and enhance pain experiences, thereby fueling fear of pain and avoidance behavior (Muris et al., 2001). Research has emphasized the significance of fear-avoidance beliefs in predicting the persistence of pain, disability, and extended sick leave in people dealing with both acute and chronic pain conditions (Turk and Wilson, 2010; Gidron, 2016). It has been suggested that self-efficacy and kinesiophobia play crucial roles in how pain can result in disability among chronic low back pain people. In fact, in this population, pain self-efficacy has been identified as a more significant mediator in the relationship between pain and disability than kinesiophobia (Costal et al., 2011). Adherence to pain self-management strategies has been associated with improved pain, depression, and disability in those with disabling chronic pain (Nicholas et al., 2012). Mindfulness practices have been linked to reduced fear-avoidance beliefs and behaviors related to chronic pain (Schütze et al., 2010). Finally, self-compassion has been proposed as a buffer in pain management, associated with more adaptive coping rather than fear-avoidance (Malpus et al., 2023). Therefore, adding self-compassion meditation to treatment for chronic pain may help people confront their fears of movement more effectively and engage in life despite their pain (Wren et al., 2012).

Regarding perceived stress, there were no significant differences between the two groups at post-test, but a significant group x time interaction effect was observed at follow-up, indicating that the HYSCM group experienced a greater reduction in stress over time. It could be speculated that the intervention’s self-compassion meditation component helped cultivate coping strategies and resilience, leading to a delayed but significant reduction in perceived stress (Allen and Leary, 2010; Wang et al., 2023). This aligns with the growing body of literature highlighting the stress-reducing effects of mindfulness and self-compassion practices (Barnard and Curry, 2011; Keng et al., 2011; Neff and Germer, 2013; Khoury et al., 2015).

In terms of perceived disability, both interventions showed a decrease in disability related to chronic low back pain over time as measured by RMQ regardless of group assignment. These findings are consistent with previous research on the benefits of physical activity, including yoga, in managing CLBP (Koerich et al., 2021; Maruf et al., 2021). Indeed, Singh (2013), found that yoga can positively impact physical disabilities in individuals with intellectual disabilities. Similarly, Maruf et al. (2021) found that physical activity level and health-related quality of life decrease with increasing CLBP-related disability. Additionally, authors have highlighted that CLBP-related disability affects individuals’ social lives, careers, and families (Koerich et al., 2021). These studies suggest that the physical component of Hatha Yoga, common to both interventions, likely contributed to the improvements in physical function and disability observed in this study.

Finally, a significant improvement in positive affect and a reduction in negative affect over time in the HYSCM group were observed. These results are consistent with literature that showed improvements in emotional regulation and coping through self-compassion meditation (Lutz et al., 2008; Turner, 2012). For instance, Sirois (2015) demonstrated a positive association between self-compassion and intentions to engage in health-promoting behaviors, with positive and negative affect serving as a valuable self-regulation resource to promote health behaviors. Similarly, Matko et al. (2022) investigated the effects of different combinations of yoga components on the participants’ body awareness, emotion regulation, affectivity, self-compassion, and distress tolerance. The study found that combining meditation with physical yoga, ethical education, and both yoga and ethical education improved self-compassion. Furthermore, Hofmann et al. (2011) highlighted how compassion meditation may reduce stress-induced subjective distress, immune response, and enhance activation of brain areas involved in emotional processing and empathy. These results suggest that self-compassion meditation, as seen in the HYSCM intervention, may facilitate better emotional regulation, and reduced negative affect in individuals with CLBP.

## 5 Conclusion

Overall, our study suggests that while Hatha Yoga alone can be beneficial in managing CLBP, the combined approach of Hatha Yoga with Self-Compassion Meditation may provide additional benefits by reducing fear of movement and improving emotional well-being. These results underline the potential of holistic, mind-body interventions in managing CLBP. Nonetheless, more research is needed to confirm these findings, explore the potential underlying mechanisms, and assess the long-term effects of such interventions.

## 6 Limitation of the study

Despite the positive and encouraging results, there are limitations that it is appropriate to highlight. Firstly, the sample size may be limited because the presence of dropouts during the post-test and one month follow-up phases could potentially impact the study’s statistical power, as the final sample size analyzed in those phases may be lower than initially planned. The participants’ pre-study and follow-up physical activity levels did not investigate, but instead chose to measure BMI as an indirect measure. Finally, a lack of a pure Control group, i.e., without any interventions, does not make a placebo effect completely excludable. Overall, these data will be necessary to discern any further effects or interactions.

## Data availability statement

The raw data supporting the conclusions of this article will be made available by the authors, without undue reservation.

## Ethics statement

The studies involving humans were approved by the Ethics Committee University of Split. The studies were conducted in accordance with the local legislation and institutional requirements. The participants provided their written informed consent to participate in this study.

## Author contributions

AD: Conceptualization, Data curation, Investigation, Methodology, Project administration, Supervision, Validation, Writing – original draft, Writing – review and editing. SA: Writing – original draft, Writing – review and editing, Investigation. BM: Writing – original draft, Writing – review and editing, Data curation, Software. VB: Data curation, Software, Writing – review and editing, Fund acquisition. NB: Writing – review and editing. GK: Writing – review and editing, Conceptualization, Data curation, Methodology, Software, Supervision, Writing – original draft.

## References

[B1] Allen A. B. Leary M. R. (2010). Self-compassion, stress, and coping. *Soc. Pers. Psychol. Compass* 4 107–118. 10.1111/j.1751-9004.2009.00246.x 20686629 PMC2914331

[B2] Asiri F. Reddy R. Tedla J. ALMohiza M. Alshahrani M. Govindappa S. (2021). Kinesiophobia and its correlations with pain, proprioception, and functional performance among individuals with chronic neck pain. *PLoS One* 16:e0254262. 10.1371/journal.pone.0254262 34237105 PMC8266083

[B3] Barnard L. K. Curry J. F. (2011). Self-compassion: Conceptualizations, correlates, & interventions. *Rev. Gen. Psychol.* 15 289–303. 10.1037/a0025754

[B4] Berry M. P. Lutz J. Schuman-Olivier Z. Germer C. Pollak S. Edwards R. R. (2020). Brief self-compassion training alters neural responses to evoked pain for chronic low back pain: A pilot study. *Pain Med.* 21 2172–2185. 10.1093/pm/pnaa178 32783054 PMC7593799

[B5] Brems C. (2015). A yoga stress reduction intervention for university faculty, staff, and graduate students. *Int. J. Yoga Therap.* 25 61–77. 10.17761/1531-2054-25.1.61 26667290

[B6] Burwinkle T. Robinson J. P. Turk D. C. (2005). Fear of movement: Factor structure of the Tampa scale of kinesiophobia in patients with fibromyalgia syndrome. *J. Pain* 6 384–391. 10.1016/j.jpain.2005.01.355 15943960

[B7] Buttner M. M. Brock R. L. O’Hara M. W. Stuart S. (2015). Efficacy of yoga for depressed postpartum women: A randomized controlled trial. *Complem. Therap. Clin. Pract.* 21 94–100. 10.1016/j.ctcp.2015.03.003 25886805

[B8] Costal L. da C. M. Maherl C. G. McAuleyl J. H. Hancockl M. J. Smeetsl R. J. E. M. (2011). Self-efficacy is more important than fear of movement in mediating the relationship between pain and disability in chronic low back pain. *Eur. J. Pain* 15 213–219. 10.1016/j.ejpain.2010.06.014 20655254

[B9] Cramer H. Lauche R. Haller H. Dobos G. (2013). A systematic review and meta-analysis of yoga for low back pain. *Clin. J. Pain* 29 450–460. 10.1097/AJP.0b013e31825e1492 23246998

[B10] Crofford L. (2015). Psychological aspects of chronic musculoskeletal pain. *Best Pract. Res. Clin. Rheumatol.* 29 147–155. 10.1016/j.berh.2015.04.027 26267008 PMC5061342

[B11] Cypress B. (1983). Characteristics of physician visits for back symptoms: A national perspective. *Am. J. Public Health* 73 389–395. 10.2105/ajph.73.4.389 6219588 PMC1650784

[B12] Dael N. Mortillaro M. Scherer K. (2012). Emotion expression in body action and posture. *Emotion* 12 1085–1081. 10.1037/a0025737 22059517

[B13] Davis K. Goodman S. Leiferman J. Taylor M. Dimidjian S. (2015). A randomized controlled trial of yoga for pregnant women with symptoms of depression and anxiety. *Complement. Ther. Clin. Pract.* 21 166–172. 10.1016/j.ctcp.2015.06.005 26256135

[B14] De Giorgio A. Dante A. Cavioni V. Padovan A. Rigonat D. Iseppi F. (2017a). The IARA model as an integrative approach to promote autonomy in COPD patients through improvement of self-efficacy beliefs and illness perception: A mixed-method pilot study. *Front. Psychol.* 8:1682. 10.3389/fpsyg.2017.01682 29062286 PMC5640890

[B15] De Giorgio A. Loscalzo R. Ponte M. Padovan A. Graceffa G. Gulotta F. (2017b). An innovative mindfulness and educational care approach in an adult patient affected by gastroesophageal reflux: The IARA model. *J. Complement. Integr. Med.* 14:154. 10.1515/jcim-2016-0154 28731313

[B16] De Giorgio A. Padulo J. Kuvačić G. (2018a). Effectiveness of yoga combined with back school program on anxiety, kinesiophobia and pain in people with non-specific chronic low back pain: A prospective randomized trial. *Muscle Lig. Tendons J.* 8 104–112. 10.11138/MLTJ/2018.8.1.104

[B17] De Giorgio A. Kuvačić G. Milić M. Padulo J. (2018b). The brain and movement: How physical activity affects the brain. *Mont. J. Sports Sci. Med.* 7 63–68. 10.26773/mjssm.180910

[B18] Delitto A. George S. Van Dillen L. Whitman J. Sowa G. Shekelle P. (2012). Low back pain. *J. Orthop. Sports Phys. Ther.* 42 A1–A57. 10.2519/jospt.2012.42.4.A1 22466247 PMC4893951

[B19] Faul F. Erdfelder E. Lang A.-G. Buchner A. (2007). G*Power 3: A flexible statistical power analysis program for the social, behavioral, and biomedical sciences. *Behav. Res. Methods* 39 175–191. 17695343 10.3758/bf03193146

[B20] Freburger J. Holmes G. Agans R. Jackman A. Darter J. Wallace A. (2009). The rising prevalence of chronic low back pain. *Arch. Intern. Med.* 169 251–258. 10.1001/archinternmed.2008.543 19204216 PMC4339077

[B21] Fritz J. M. George S. Z. Delitto A. (2001). The role of fear-avoidance beliefs in acute low back pain: Relationships with current and future disability and work status. *Pain* 94 7–15. 10.1016/S0304-3959(01)00333-5 11576740

[B22] Furunes H. Storheim K. Brox J. Johnsen L. Skouen J. Franssen E. (2017). Total disc replacement versus multidisciplinary rehabilitation in patients with chronic low back pain and degenerative discs: 8-year follow-up of a randomized controlled multicenter trial. *Spine J.* 17 1480–1488. 10.1016/j.spinee.2017.05.011 28583869

[B23] Gidron Y. (2016). *Encyclopedia of Behavioral Medicine.* New York: Springer, 10.1007/978-1-4614-6439-6_1131-2

[B24] Hofmann S. Grossman P. Hinton D. (2011). Loving-kindness and compassion meditation: Potential for psychological interventions. *Clin. Psychol. Rev.* 31 1126–1132. 10.1016/j.cpr.2011.07.003 21840289 PMC3176989

[B25] Hopkins W. Marshall S. Batterham A. Hanin J. (2009). Progressive statistics for studies in sports medicine and exercise science. *Med. Sci. Sports Exerc.* 41 3–13. 10.1249/MSS.0b013e31818cb278 19092709

[B26] Indahl A. (2004). Low back pain: Diagnosis, treatment, and prognosis. *Scand. J. Rheumatol.* 33 199–209. 10.1080/03009740410006916 15370713

[B27] Keng S.-L. Smoski M. J. Robins C. J. (2011). Effects of mindfulness on psychological health: A review of empirical studies. *Clin. Psychol. Rev.* 31 1041–1056. 10.1016/j.cpr.2011.04.006 21802619 PMC3679190

[B28] Kent P. M. Keating J. L. (2005). The epidemiology of low back pain in primary care. *Chiropract. Osteopathy* 13:13. 10.1186/1746-1340-13-13 16045795 PMC1208926

[B29] Khoury B. Sharma M. Rush S. Fournier C. (2015). Mindfulness-based stress reduction for healthy individuals: A meta-analysis. *J. Psychosom. Res.* 78 519–528. 10.1016/j.jpsychores.2015.03.009 25818837

[B30] Koerich M. H. A. Meirelles B. H. S. Echevaría-Guanilo M. E. Danielewicz A. L. Schwertner D. S. Knabben R. J. (2021). Disability in people with chronic low back pain treated in primary care. *Fisioter. Em. Movimento* 34:34121. 10.1590/fm.2021.34121

[B31] La Cour P. Petersen M. (2015). Effects of mindfulness meditation on chronic pain: A randomized controlled trial. *Pain Med.* 16 641–652. 10.1111/pme.12605 25376753

[B32] Ledford C. (2017). Spine conditions: Mechanical and inflammatory low back pain. *FP Essent.* 461 15–20. 29019640

[B33] Lee M. Moon W. Kim J. (2014). Effect of Yoga on Pain, Brain-Derived Neurotrophic Factor, and Serotonin in Premenopausal Women with Chronic Low Back Pain. *Evid. Based Complement. Altern. Med.* 2014 1–7. 10.1155/2014/203173 25120574 PMC4120477

[B34] Liang K.-Y. Zeger S. L. (1986). Longitudinal data analysis using generalized linear models. *Biometrika* 73 13–22. 10.1093/biomet/73.1.13

[B35] Lutz A. Brefczynski-Lewis J. Johnstone T. Davidson R. (2008). Regulation of the neural circuitry of emotion by compassion meditation: Effects of meditative expertise. *PLoS One* 3:e1897. 10.1371/journal.pone.0001897 18365029 PMC2267490

[B36] Lysne P. Palit S. Morais C. DeMonte L. Lakdawala M. Sibille K. (2021). Adaptability and Resilience in Aging Adults (ARIAA): Protocol for a pilot and feasibility study in chronic low back pain. *Pilot Feasibility Stud.* 7:188. 10.1186/s40814-021-00923-y 34666839 PMC8525058

[B37] Madsen A. Sharififar S. Oberhaus J. Vincent K. Vincent H. (2022). Anxiety state impact on recovery of runners with lower extremity injuries. *PLoS One* 17:e0278444. 10.1371/journal.pone.0278444 36454920 PMC9714898

[B38] Malpus Z. Nazar Z. Smith C. Armitage L. (2023). Compassion focused therapy for pain management: ‘3 systems approach’ to understanding why striving and self-criticism are key psychological barriers to regulating activity and improving self-care for people living with persistent pain. *Br. J. Pain* 17 87–102. 10.1177/20494637221133630 36815069 PMC9940251

[B39] Maruf F. A. Ani K. Akosile C. O. Nwodo O. D. Okonkwo P. U. Awhen P. A. (2021). Correlates and predictors of disability related to chronic low-back pain in a Nigerian population. *J. Musculoskeletal Res.* 24:4. 10.1142/S0218957721500111

[B40] Matko K. Sedlmeier P. Bringmann H. C. (2022). Embodied Cognition in Meditation, Yoga, and Ethics—An Experimental Single-Case Study on the Differential Effects of Four Mind–Body Treatments. *Int. J. Environ. Res. Public Health* 19:11734. 10.3390/ijerph191811734 36142006 PMC9517053

[B41] Mondo M. Sechi C. Cabras C. (2021). Psychometric evaluation of three versions of the Italian Perceived Stress Scale. *Curr. Psychol.* 40 1884–1892. 10.1007/s12144-019-0132-8

[B42] Monticone M. Giorgi I. Baiardi P. Barbieri M. Rocca B. Bonezzi C. (2010). Development of the Italian version of the Tampa Scale of Kinesiophobia (TSK-I): Cross-cultural adaptation, factor analysis, reliability, and validity. *Spine* 35 1241–1246. 10.1097/BRS.0b013e3181bfcbf6 20216338

[B43] Muris P. Vlaeyen J. W. S. Meesters C. Vertongen S. (2001). Anxiety sensitivity and fear of pain in children. *Percept. Motor Skills* 92 456–458. 10.2466/pms.2001.92.2.456 11361307

[B44] Neff K. D. Germer C. K. (2013). A pilot study and randomized controlled trial of the mindful self-compassion program. *J. Clin. Psychol.* 69 28–44. 10.1002/jclp.21923 23070875

[B45] Nicholas M. Asghari A. Corbett M. Smeets R. Wood B. Overton S. (2012). Is adherence to pain self-management strategies associated with improved pain, depression and disability in those with disabling chronic pain? *Eur. J. Pain* 16 93–104. 10.1016/j.ejpain.2011.06.005 21705246

[B46] Padovan A. Kuvačić G. Gulotta F. Sellami M. Bruno C. Isoardi M. (2018). A new integrative approach to increase quality of life by reducing pain and fear of movement in patients undergoing total hip arthroplasty: The IARA model. *Psychol. Health Med.* 23 1223–1230. 10.1080/13548506.2018.1488080 29944000

[B47] Padovan A. M. Oprandi G. Padulo J. Bruno C. Isoardi M. Gulotta F. (2019). A novel integrative approach to improve the quality of life by reducing pain and kinesiophobia in patients undergoing TKA: The IARA Model. *Muscle Lig. Tendons J.* 8:93. 10.32098/mltj.01.2018.12

[B48] Padua R. Padua L. Ceccarelli E. Romanini E. Zanoli G. Bondì R. (2002). Italian version of the Roland Disability Questionnaire, specific for low back pain: Cross-cultural adaptation and validation. *Eur. Spine J.* 11 126–129. 10.1007/s005860100262 11956918 PMC3610499

[B49] Pascoe M. Thompson D. Jenkins Z. Ski C. (2017). Mindfulness mediates the physiological markers of stress: Systematic review and meta-analysis. *J. Psychiatr. Res.* 95 156–178. 10.1016/j.jpsychires.2017.08.004 28863392

[B50] Petrucci G. Papalia G. Russo F. Vadalà G. Piredda M. De Marinis M. (2021). Psychological approaches for the integrative care of chronic low back pain: A systematic review and metanalysis. *Int. J. Environ. Res. Public Health* 19:60. 10.3390/ijerph19010060 35010319 PMC8751135

[B51] Posadzki P. Ernst E. (2011). Yoga for low back pain: a systematic review of randomized clinical trials. *Clin. Rheumatol.* 30 1257–1262. 10.1007/s10067-011-1764-8 21590293

[B52] Ramaci T. Rapisarda V. Bellini D. Mucci N. De Giorgio A. Barattucci M. (2020). Mindfulness as a protective factor for dissatisfaction in HCWs: The moderating role of mindful attention between climate stress and job satisfaction. *Int. J. Environ. Res. Public Health* 17:3818. 10.3390/ijerph17113818 32481543 PMC7312809

[B53] Reiner K. Tibi L. Lipsitz J. (2013). Do mindfulness-based interventions reduce pain intensity? A critical review of the literature. *Pain Med.* 14 230–242. 10.1111/pme.12006 23240921

[B54] Russo M. Deckers K. Eldabe S. Kiesel K. Gilligan C. Vieceli J. (2018). Muscle control and non-specific chronic low back pain. *Neuromodulation* 21 1–9. 10.1111/ner.12738 29230905 PMC5814909

[B55] Sabnis A. B. Chamoli U. Diwan A. D. (2017). Is L5-S1 motion segment different from the rest? A radiographic kinematic assessment of 72 patients with chronic low back pain. *Eur. Spine J.* 10.1007/s00586-017-5400-4 [Epub ahead of print]. 29181575

[B56] Schütze R. Rees C. Preece M. Schütze M. (2010). Low mindfulness predicts pain catastrophizing in a fear-avoidance model of chronic pain. *Pain* 148 120–127. 10.1016/j.pain.2009.10.030 19944534

[B57] Sherman K. J. Wellman R. D. Cook A. J. Cherkin D. C. Ceballos R. M. (2013). Mediators of yoga and stretching for chronic low back pain. *Evid. Based Complement. Altern. Med.* 2013 1–11. 10.1155/2013/130818 23690832 PMC3652191

[B58] Singh S. (2013). Intellectual disabilities and yoga. *Int. J. Yoga* 6 80–81. 10.4103/0973-6131.105954 23436969 PMC3573550

[B59] Sirois F. (2015). A self-regulation resource model of self-compassion and health behavior intentions in emerging adults. *Prev. Med. Rep.* 2 218–222. 10.1016/j.pmedr.2015.03.006 26844074 PMC4721380

[B60] Staes F. Stappaerts K. Lesaffre E. Vertommen H. (2007). Low back pain in Flemish adolescents and the role of perceived social support and effect on the perception of back pain. *Acta Paediatr.* 92 444–451. 10.1111/j.1651-2227.2003.tb00576.x 12801111

[B61] Tekur P. Singphow C. Nagendra H. R. Raghuram N. (2008). Effect of short-term intensive yoga program on pain, functional disability and spinal flexibility in chronic low back pain: A randomized control study. *J. Altern. Complement. Med.* 14 637–644. 10.1089/acm.2007.0815 18673078

[B62] Terracciano A. McCrae R. R. Costa P. T. (2003). Factorial and construct validity of the Italian Positive and Negative Affect Schedule (PANAS). *Eur. J. Psychol. Assess.* 19 131–141. 10.1027//1015-5759.19.2.131 20467578 PMC2868265

[B63] Trocoli T. O. Botelho R. V. (2016). Prevalence of anxiety, depression and kinesiophobia in patients with low back pain and their association with the symptoms of low back spinal pain. *Rev. Brasil. Reumatol.* 56 330–336. 10.1016/j.rbre.2016.02.010 27476626

[B64] Turk D. C. Wilson H. D. (2010). Fear of pain as a prognostic factor in chronic pain: Conceptual models, assessment, and treatment implications. *Curr. Pain Headache Rep.* 14 88–95. 10.1007/s11916-010-0094-x 20425197 PMC2872063

[B65] Turner R. (2012). The need for systematic ethnopsychology: The ontological status of mentalistic terminology. *Anthropol. Theory* 12 29–42. 10.1177/1463499612436462

[B66] Van Zundert J. Van Boxem K. Vanelderen P. Puylaert M. De Vooght P. Mestrum R. (2013). Establishing the diagnosis of low back pain: Patient selection for interventional pain medicine. *Pain Manag.* 3 129–136. 10.2217/pmt.13.3 24645997

[B67] Veehof M. M. Trompetter H. R. Bohlmeijer E. T. Schreurs K. M. G. (2016). Acceptance- and mindfulness-based interventions for the treatment of chronic pain: a meta-analytic review. *Cogn. Behav. Therapy* 45 5–31. 10.1080/16506073.2015.1098724 26818413

[B68] Viglino F. Sellami M. Broglio F. Scuntero P. Padovan A. Maulini C. (2022). The role of the person focused IARA model in reducing anxiety and improving body awareness and illness management in diabetics with acquired lipodystrophy: A Mixed-Method Study. *J. Pers. Med.* 12:1865. 10.3390/jpm12111865 36579585 PMC9695520

[B69] Vos T. Barber R. M. Bell B. Bertozzi-Villa A. Biryukov S. Bolliger I. (2015). Global, regional, and national incidence, prevalence, and years lived with disability for 301 acute and chronic diseases and injuries in 188 countries, 1990-2013: a systematic analysis for the Global Burden of Disease Study 2013. *Lancet* 386 743–800. 10.1016/S0140-6736(15)60692-4 26063472 PMC4561509

[B70] Wand B. Parkitny L. O’Connell N. Luomajoki H. McAuley J. Thacker M. (2011). Cortical changes in chronic low back pain: current state of the art and implications for clinical practice. *Man Ther.* 16 15–20. 10.1016/j.math.2010.06.008 20655796

[B71] Wang R. Gu X. Zhang Y. Luo K. Zeng X. (2023). Loving-kindness and compassion meditations in the workplace: A meta-analysis and future prospects. *Stress Health* 10.1002/smi.3273 [Epub ahead of print]. 37221984

[B72] Will J. Bury D. Miller J. (2018). Mechanical low back pain. *Am. Fam. Phys.* 98 421–428. 30252425

[B73] Wren A. Somers T. Wright M. Goetz M. Leary M. Fras A. (2012). Self-compassion in patients with persistent musculoskeletal pain: Relationship of self-compassion to adjustment to persistent pain. *J. Pain Symptom Manage.* 43 759–770. 10.1016/j.jpainsymman.2011.04.014 22071165

